# Protective Effects of a *Rhodiola Crenulata* Extract and Salidroside on Hippocampal Neurogenesis against Streptozotocin-Induced Neural Injury in the Rat

**DOI:** 10.1371/journal.pone.0029641

**Published:** 2012-01-03

**Authors:** Ze-qiang Qu, Yan Zhou, Yuan-shan Zeng, Yu-kun Lin, Yan Li, Zhi-qiang Zhong, Wood Yee Chan

**Affiliations:** 1 Division of Neuroscience, Department of Histology and Embryology, Zhongshan School of Medicine, Sun Yat-sen University, Guangzhou, China; 2 Key Laboratory for Stem Cells and Tissue Engineering, Sun Yat-sen University, Ministry of Education, Guangzhou, China; 3 School of Biomedical Sciences, Faculty of Medicine, The Chinese University of Hong Kong, Hong Kong, China; Case Western Reserve University, United States of America

## Abstract

Previously we have demonstrated that a *Rhodiola crenulata* extract *(RCE)*, containing a potent antioxidant salidroside, promotes neurogenesis in the hippocampus of depressive rats. The current study was designed to further investigate the protective effect of the *RCE* on neurogenesis in a rat model of Alzheimer's disease (AD) induced by an intracerebroventricular injection of streptozotocin (STZ), and to determine whether this neuroprotective effect is induced by the antioxidative activity of salidroside. Our results showed that pretreatment with the *RCE* significantly improved the impaired neurogenesis and simultaneously reduced the oxidative stress in the hippocampus of AD rats. *In vitro* studies revealed that (1) exposure of neural stem cells (NSCs) from the hippocampus to STZ strikingly increased intracellular reactive oxygen species (ROS) levels, induced cell death and perturbed cell proliferation and differentiation, (2) hydrogen peroxide induced similar cellular activities as STZ, (3) pre-incubation of STZ-treated NSCs with catalase, an antioxidant, suppressed all these cellular activities induced by STZ, and (4) likewise, pre-incubation of STZ-treated NSCs with salidroside, also an antioxidant, suppressed all these activities as catalase: reduction of ROS levels and NSC death with simultaneous increases in proliferation and differentiation. Our findings indicated that the *RCE* improved the impaired hippocampal neurogenesis in the rat model of AD through protecting NSCs by its main ingredient salidroside which scavenged intracellular ROS.

## Introduction

Alzheimer's disease (AD) is an irreversible neurodegenerative disorder of the brain characterized by the progressive cognitive decline with a poor outcome and unknown etiology. Neuropathologically, AD is defined by an accumulation of extracellular senile plaques and intracellular neurofibrillary tangles, regionalized neuronal death and loss of synaptic connections within selective brain regions. It has been proposed that oxidative stress and dysfunction of neurogenesis play important roles in the pathogenesis of AD [1], [2].

It has already been shown that neurogenesis occurs in the adult mammalian brain and plays roles in both learning and memory processes and also recovery from injury [3], [4]. Abnormalities in neurogenesis may lead to disorders of learning and memory in humans such as AD [5]. Studies indicated that many AD risk factors which are associated with cognitive impairments also significantly affect hippocampal neurogenesis [3], [4], [6]. In various rodent models of AD, including mice with mutation in amyloid precursor protein or presenilin 1, severe impairment of neurogenesis in the subgranular zone of the dentate gyrus has been reported [6], [7]. In the hippocampus of patients with AD, a compensatory enhancement of neurogenesis has been observed, but this enhanced neurogenesis is not able to compensate for severe neuronal loss [2], [8]. The therapeutic effects of some AD drugs have also been ascribed to their ability to increase cerebral neurogenesis both *in vitro* and *in vivo* [9]. All these findings suggest that impaired neurogenesis may attribute to the pathogenesis of AD. Measures to enhance neurogenesis therefore have huge therapeutic potentials, and abnormal neurogenesis is a promising therapeutic target for this disease [10].

Mounting evidence has also indicated the involvement of oxidative stress in the pathogenesis of the disease. It has been shown that free radicals and oxidative stress induce memory deficits and hence behavioral impairments in AD patients [11], [12]. The reactive oxygen species (ROS), which are formed during oxidative stress, induces cellular and molecular abnormalities in sporadic AD [13]. Although the exact mechanisms underlying these deleterious effects remain unclear, it has already been known that oxidative stress occurs before the formation of neurofibrillary tangles and senile plaques, both of which are hallmarks of AD [14], [15]. An accumulation of free radicals as a result of an imbalance between their production and removal by the antioxidant system may lead to increases of lipid and protein peroxidation, DNA oxidation and calcium dysregulation, all of which eventually lead to neuronal cell death [16], [17]. Importantly, oxidative stress has also been considered to be able to affect neurogenesis [18]. Environmental stresses including exposure to ROS have been shown to inhibit neurogenesis and are associated with the onset of cognitive impairments [19], [20]. Consumption of potent antioxidants, e.g. melatonin and polyunsaturated fatty acids, could have a significant effect in reducing the decline of neurogenesis and attenuating the impairment of cognitive functions [21], [22].

Medicinal plant *Rhodiola crenulata (R. crenulata)* grows at high altitudes in the Arctic and mountainous regions, and is commonly used in phytotherapy in China, Uzbekistan and other Asian countries. It has been known to be able to stimulate the nervous system, alleviate depression, enhance work performance, eliminate fatigue and prevent altitude sickness [23]. Extracts of *R. crenulata* have been shown to possess stress-protective and anti-oxidative activities, and ingestion of the plant extracts from the genus *Rhodiola* may improve cognitive functions [24], [25], reduce mental fatigue [26], mitigate free radicals and oxidative insults [27]–[29] and enhance neuroprotective [24] and anti-depressive activities [30]. Phytochemical investigations revealed that the *R. crenulata* root contains about 21 compounds. Salidroside (rhodioloside), rosavins and p-tyrosol are thought to be the most important constituents for the therapeutic activities of the plant [31], [32]. Among these, salidroside has been found to have marked antioxidant effects and its activity in scavenging superoxide radicals is concentration- and time-dependent [33]. Salidroside also has protective effects against hydrogen peroxide-induced apoptosis in SH-SY5Y human neuroblastoma cells [34].

Although the medicinal plant genus *Rhodiola* has been known to have significant anti-oxidative and neuroprotective properties, there are no reports on its effects on the neurogenesis in AD, and much remains unknown about its action mechanism and active ingredients. The aim of this work was to determine whether the impaired hippocampal neurogenesis in a rat model of AD induced by the intracerebroventricular (ICV) injection of streptozotocin (STZ) can be rescued by the pre-treatment with a *R. crenulata* extract (*RCE*), and to determine whether salidroside, a main ingredient of the *RCE* which protects neural stem cells (NSCs) by scavenging ROS, contributes to the protective effect of the *RCE* on neurogenesis.

## Results

### In Vivo Studies

To study the protective effects of *R. crenulata* on AD, an alcohol extract (*RCE*) was first prepared, and adult rats were then treated with the *RCE* by gavage everyday for three weeks before AD was induced by bilateral stereotactic injections of streptozotocin to both sides of the cerebral ventricles. It was found that pre-treatment of the *RCE* resulted in enhanced neurogenesis and decreased oxidative stress in the hippocampus of AD rats.

#### (i) *RCE* Enhanced Neurogenesis in the Hippocampus of STZ-treated Rats

**Figure 1:**
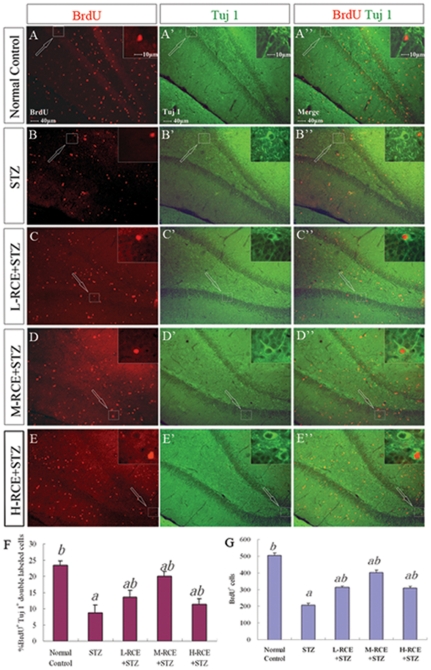
BrdU/Tuj1 double immunofluorescence labeling in the rat hippocampus. (A–E, A′–E′, A″–E″) Representative photomicrographs of BrdU (red fluorescence in the nuclei, A–E) and Tuj1 labeling (green fluorescence in the cytoplasm, A′–E′; A″–E″ are merged images of BrdU and Tuj1 labeling) from different experimental groups: (A–A″) Normal control group, (B–B″) streptozotocin-treated (STZ) group, (C–C″) Low dose of *RCE* pre-treatment followed by STZ treatment group (L-RCE+STZ), (D–D″) Medium dose of *RCE* pre-treatment followed by STZ treatment group (M-RCE+STZ) and (E–E″) High dose of *RCE* pre-treatment followed by STZ treatment group (H-RCE+STZ). The upper right inset of the photomicrograph is the enlarged view of a double-labeled cell in the boxed area (arrow). (F) Bar chart showing the percentages of differentiating neurons (BrdU^+^Tuj1^+^/BrdU^+^×100%). (G) Bar chart showing BrdU-positive cell counts. Values are expressed as mean±SD. a: *p*<0.05 compared with the normal control group; b: *p*<0.05 compared with the STZ group.

To assess neurogenesis, brain sections through the hippocampus labeled with BrdU were double-stained with BrdU (proliferation marker) and Tuj1 (immature neuronal marker) antibodies. In rats only treated with STZ, both the percentage of differentiating neurons (BrdU^+^Tuj1^+^ double positive cells amongst all BrdU^+^ cells, Figure 1B-B″ and F) and the total number of dividing cells (BrdU^+^ cells, Figure 1B-B″ and G) were significantly decreased, as compared with those for the normal control group (*p*<0.05) (Figure 1A-A″, F and G). These decreases were however significantly reduced by the pre-treatment with different doses of the *RCE* when compared to the STZ-treated group (*p*<0.05) (Figure 1B-B″, C-C″, D-D″, E-E″, F and G), although a complete restoration to the normal control levels (without STZ treatment) of neurogenesis was not achieved (Figure 1F and G). Our findings indicated that neurogenesis in the STZ-treated rats was decreased and this decrease could be partially rescued by the pre-treatment with the *RCE*.

#### (ii) *RCE* Reduced Oxidative Stress in the Hippocampus of STZ-treated Rats

**Figure 2:**
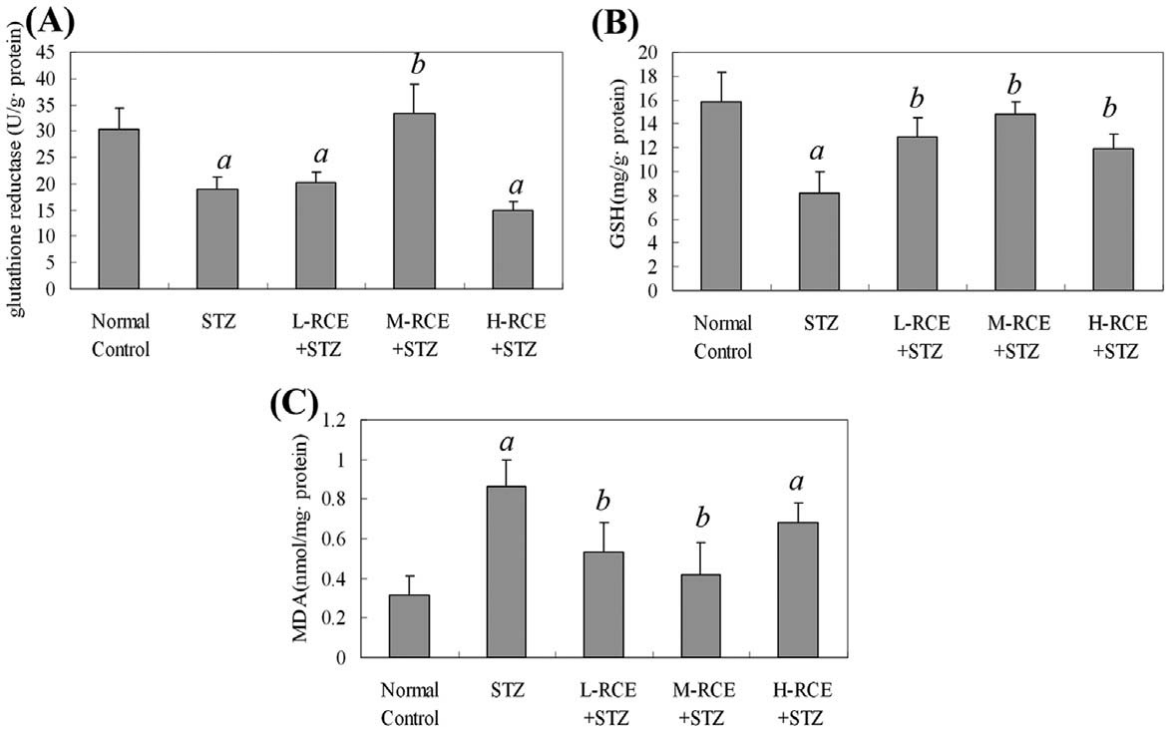
Oxidative stress in the hippocampus of STZ-treated rats. (A) Activities of glutathione reductase (GR). (B) Amounts of reduced glutathione (GSH). (C) Amounts of malondialdehyde (MDA). Values are expressed as means±SD. a: *p*<0.05 compared with the normal control group; b: *p*<0.05 compared with the STZ group. Normal Control: normal control group; STZ: streptozotocin-treated group; L-RCE+STZ, M-RCE+STZ and H-RCE+STZ: pre-treatment groups with low, medium and high concentrations of the *R. crenulata* extract *(RCE)*, respectively, followed by STZ treatment.

To assess oxidative stress in the hippocampus, we measured spectrophotometrically both the activity of glutathione reductase (GR) and the amount of reduced glutathione (GSH) as the measurements for the activity of glutathione antioxidant system. We also measured the amount of malondialdehyde (MDA) as the indicator of the level of lipid peroxidation in the hippocampal tissue. We found that the activity of the glutathione antioxidant system as reflected by the activity of GR and the amount of GSH was markedly decreased in STZ-treated rats as compared to the normal control group (*p*<0.05) (Figure 2A and B), and these decreases were accompanied by a significant increase in the level of lipid peroxidation as indicated by the increased amount of MDA (*p*<0.05) (Figure 2C). Pre-treatment with different doses of the *RCE* for 3 weeks before the induction of AD by STZ significantly reduced the decrease of the activity of the glutathione system and suppressed the elevation of MDA in STZ-treated rats, especially in the group pre-treated with a medium dose of the *RCE* (M-RCE) as compared to the STZ group (*p*<0.05) (Figure 2A, B and C). The results demonstrated a prominent anti-oxidative effect of the *RCE* on the hippocampus of the rat model of AD induced by STZ injections.

### In Vitro Studies

**Figure 3:**
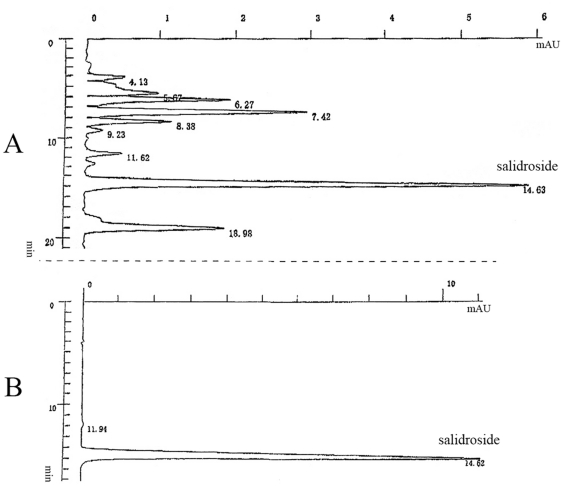
Analytical HPLC chromatograms. (A) *R. crenulata* extract (*RCE*); (B) salidroside control. Note that salidroside is the most abundant ingredient of the *RCE*.

**Figure 4:**
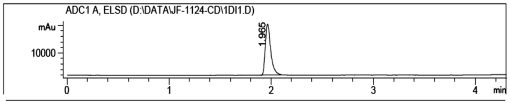
HPLC chromatogram of salidroside. Note that the preparation of salidroside is 100% pure.

Next, we sought to determine the cellular mechanism underlying the protective activities of the *RCE* against the impaired neurogenesis in the hippocampus of STZ-treated rats. We first found that salidroside was the main ingredient of the *RCE* (Figure 3) and subsequently procured a pure preparation of salidroside (Figure 4) for the studies of the effects of salidroside on neural stem cells (NSCs) from the hippocampus *in vitro*.

#### (i) Optimal Doses of STZ and Salidroside for NSC Culture

**Figure 5:**
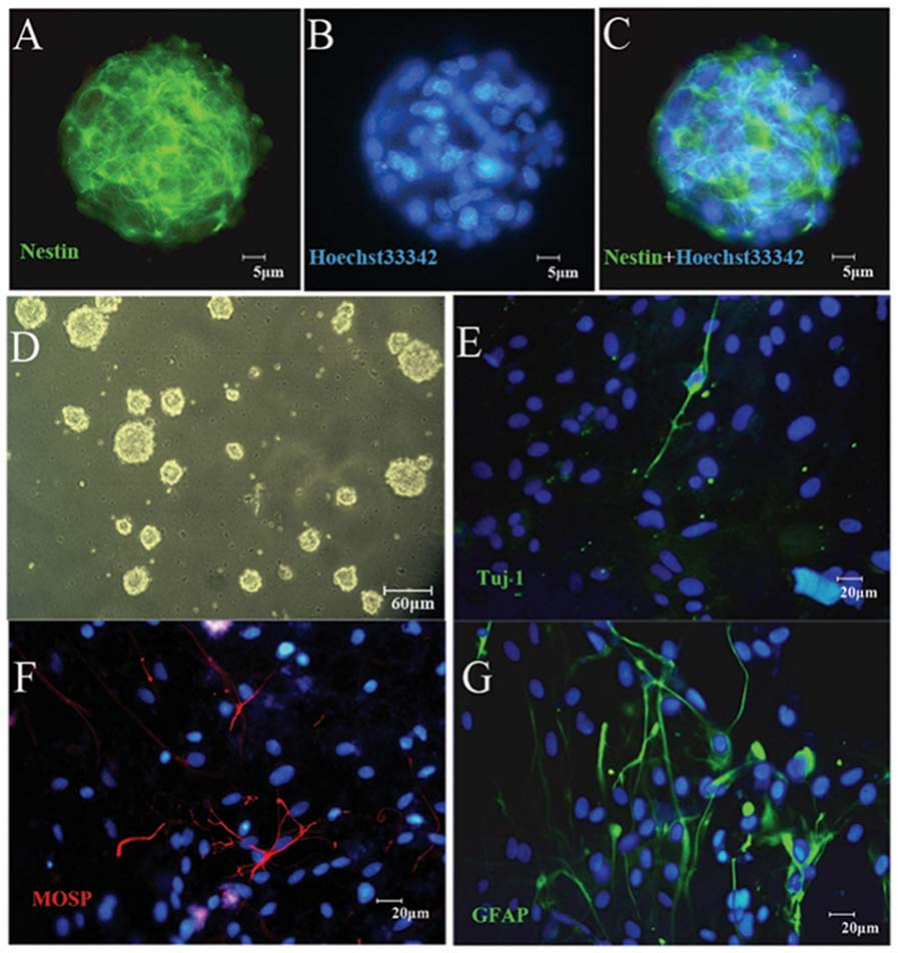
Neurosphere culture derived from the rat hippocampus. (A–C) Neurosphere at the second passage is immunoreactive to nestin, a marker of neural stem cells (green), and counterstained by nuclear fluorescent stain Hoechst33342 (blue). (D) Hippocampal cells from newborn rats proliferated and aggregated to form neurospheres one week after cultured in the DMEM/F-12 serum-free medium supplemented with B27 and bFGF. (E–G) Cells from the neurospheres show positive immunoreactivity to Tuj1 (E), MOSP (F), and GFAP (G) (markers of differentiating neurons, oligodendrocytes and astroglia, respectively) one week after cultured in the differentiation medium (DMEM/F12+10% FBS).

Hippocampal cells of newborn rats were isolated and cultured in DMEM/F-12 serum-free medium supplemented with B27 and bFGF. One week after culture, they aggregated and formed spheroid neurospheres (Figure 5A–C, D). Immunofluorescence staining revealed that cells within neurospheres were immunoreactive to nestin (marker of neural stem cells) (Figure 5A–C). Neurospheres continued to grow and were passaged once a week. When a culture medium with 10% fetal bovine serum but without bFGF (i.e. differentiation medium) and an adhesive culture surface were used, cells derived from the neurospheres became immunoreactive to Tuj1 (immature neuronal marker) (Figure 5E), myelin oligodendrocyte specific protein (MOSP, oligodendrocyte marker) (Figure 5F) and glial fibrillary acid protein (GFAP, astrocyte marker) (Figure 5G). These observations suggested that neurospheres derived from the hippocampus exhibited active proliferation, self-renewal (i.e. continuous growth of neurospheres and cell divisions within neurospheres for many passages) and multipotent properties *in vitro*.

**Figure 6:**
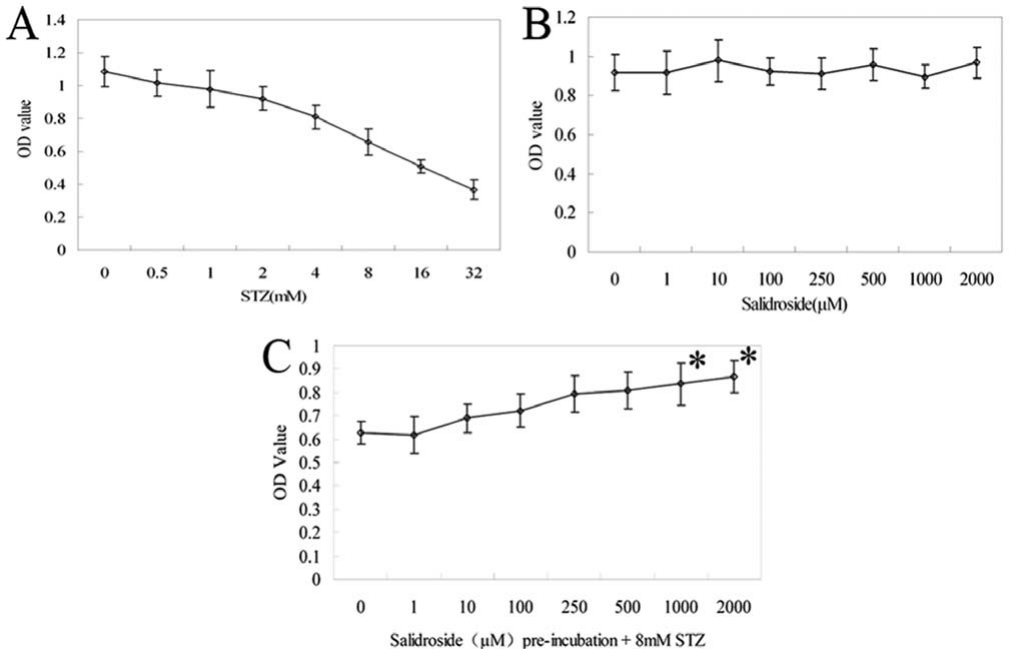
Viability of NSCs, as assayed by the MTT, at various doses of STZ and salidroside. (A) Cells from neurospheres at the second passage were incubated with various concentrations of STZ for 4 hr. STZ exhibits a dose-dependent cytotoxic effect on NSCs. (B) Salidroside at a concentration up to 2 mM for 12 hr shows no cytotoxic effects on NSCs. (C) Cells from neurospheres were pre-incubated with various concentrations of salidroside for 12 hr before exposure to 8 mM STZ. Salidroside at 1 or 2 mM exhibits significant protection against STZ-induced cytotoxity on NSCs. ^*^ *p*<0.05 compared with the control value without salidroside. Values are expressed as means±SD.

Our *in vivo* studies indicated that the intracerebroventricular injection of STZ to rats impaired neurogenesis and induced excessive oxidative stress in the hippocampus. It is thus speculated that STZ exerted a cytotoxic effect through oxidative damages on neural stem cells (NSCs) which are thought to be important for neurogenesis within the hippocampus. To determine the cytotoxicity of STZ and its optimum dose for the subsequent experiments, NSCs were treated with increasing concentrations of STZ for 4 hours and examined with the MTT assay (Figure 6A). A dose-response curve for the cytotoxicity of STZ on NSCs was obtained, and the IC_50_ of STZ was found to be 10.79 mM (calculated with Formula two, see Materials and Methods). Considering the mild protective effects of salidroside, we chose a concentration of 8 mM STZ for all subsequent experiments.

Previous studies revealed that salidroside protected neuronal cells against apoptosis induced by oxidative stress [34] and hypoglycemia and cytotoxicity induced by low concentrations of serum [35], but the effects of salidroside on undifferentiated cells, particularly NSCs, have not been examined. To this end, we incubated hippocampus-derived NSCs with increasing concentrations of salidroside and monitored the viability of the cells with the MTT assay. Our findings showed that direct incubation with salidroside up to a concentration of 2 mM did not affect the viability of NSCs (Figure 6B), but when the cells were pre-incubated with various concentrations of salidroside for 12 hours and then subject to 8 mM STZ for 4 hours, we found that the viability of NSCs was significantly higher at higher pre-incubation concentrations of salidroside (Figure 6C), suggesting that pre-incubation with salidroside protected NSCs against STZ-induced injury. Since salidroside at 1 mM was shown to significantly increase cell viability (Figure 6C), the concentration of 1 mM salidroside was chosen for the subsequent experiments.

With the MTT assay, we demonstrated in the aforementioned experiments that the 12-hour pre-incubation with 1 mM of salidroside significantly protected NSCs from the cytotoxicity induced by the 4-hour treatment of 8 mM of STZ. Based on this information, we then performed a full survey on the effects of salidroside on NSCs following STZ-induced injury.

#### (ii) STZ Decreased NSC Survival, Proliferation and Neuronal Differentiation but Increased ROS

**Figure 7:**
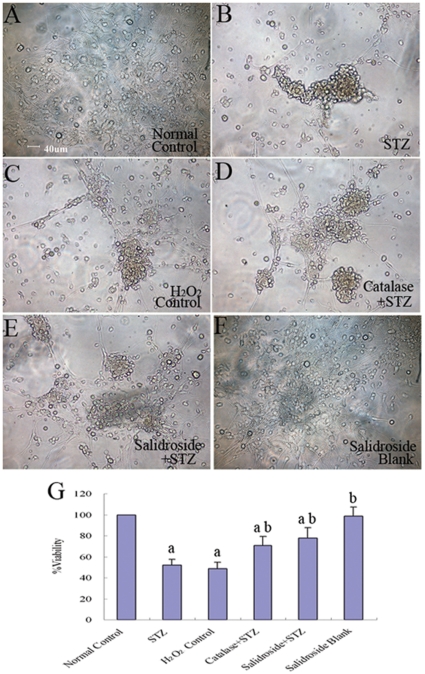
Cell morphology (A–F) and cell viability as measured by the MTT assay (G). NSCs at the second passage were seeded on poly-L-lysine-coated culture plates and grouped for the following treatments: (A) Normal control group without any treatment; (B) STZ group treated with STZ for four hours; (C) H_2_O_2_ control group treated with H_2_O_2_ for four hours; (D) Catalase+STZ group treated with catalase for twelve hours followed by four-hour incubation with STZ; (E) Salidroside+STZ group treated with salidroside for twelve hours followed by four-hour incubation with STZ; (F) Salidroside blank group treated with salidroside for twelve hours. Values are expressed as mean±SD. ^a^ *p*<0.05 compared with the normal control group; ^b^ *p*<0.05 compared with the STZ group.

**Figure 8:**
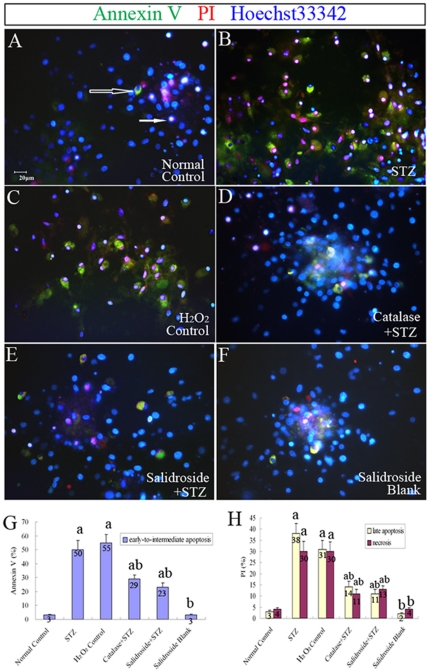
Annexin V and PI immunolabeling for detection of apoptosis and necrosis in NSCs, respectively. (A–F) Representative fluorescence photomicrographs of NSCs immunoreactive to annexin V (green) and PI (red) with their nucleus counterstained with Hoechst33342 (blue) in six experimental groups: (A) Normal control group; (B) STZ group; (C) H_2_O_2_ control group; (D) Catalase+STZ group; (E) Salidroside+STZ group; (F) Salidroside blank group. Cell at early to intermediate stages of apoptosis are positive to Annexin V but negative to PI (open arrow); late apoptosis cells are PI positive with chromatin condensation; and necrotic cells are PI positive without chromatin condensation (solid arrow). (G) Bar chart showing the percentages of annexin V-positive cells which are at the early-to-intermediate stage of apoptosis. (H) Bar chart showing the percentages of PI-positive cells, indicating that they are either late apoptotic or necrotic cells. Values are expressed as mean±SD. Numbers within bars represent the actual numerical reading on the y-axis. ^a^ *p*<0.05 compared with the normal control group; ^b^ *p*<0.05 compared with the STZ group.

**Figure 9:**
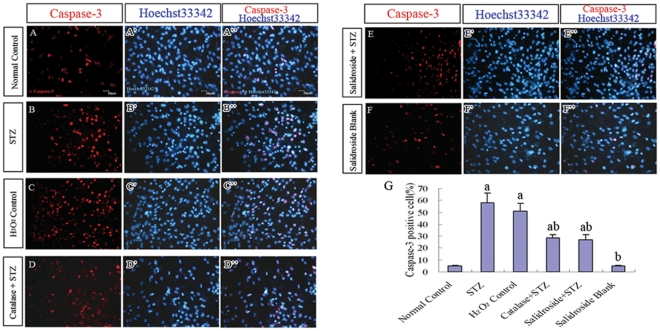
Immunofluorescence staining for cleaved caspase-3 (p17/19 active fragments) on NSCs. (A–F, A′–F′, A″–F″) Representative photomicrographs of caspase-3 immunoreactive cells (A–F) with their nuclei counterstained by the blue fluorescent nuclear stain Hoechst33342 (A′–F′) in six experimental groups: (A-A″) Normal control group, (B-B″) STZ group, (C-C″) H_2_O_2_ control group, (D-D″) Catalase+STZ group, (E-E″) Salidroside+STZ group and (F-F″) Salidroside blank group. (G) Bar chart showing the percentages of caspase-3 immunoreactive cells. Values are expressed as mean±SD. Numbers within bars represent the actual numerical reading on the y-axis. ^a^ *p*<0.05 compared with the normal control group; ^b^ *p*<0.05 compared with the STZ group.

**Figure 10:**
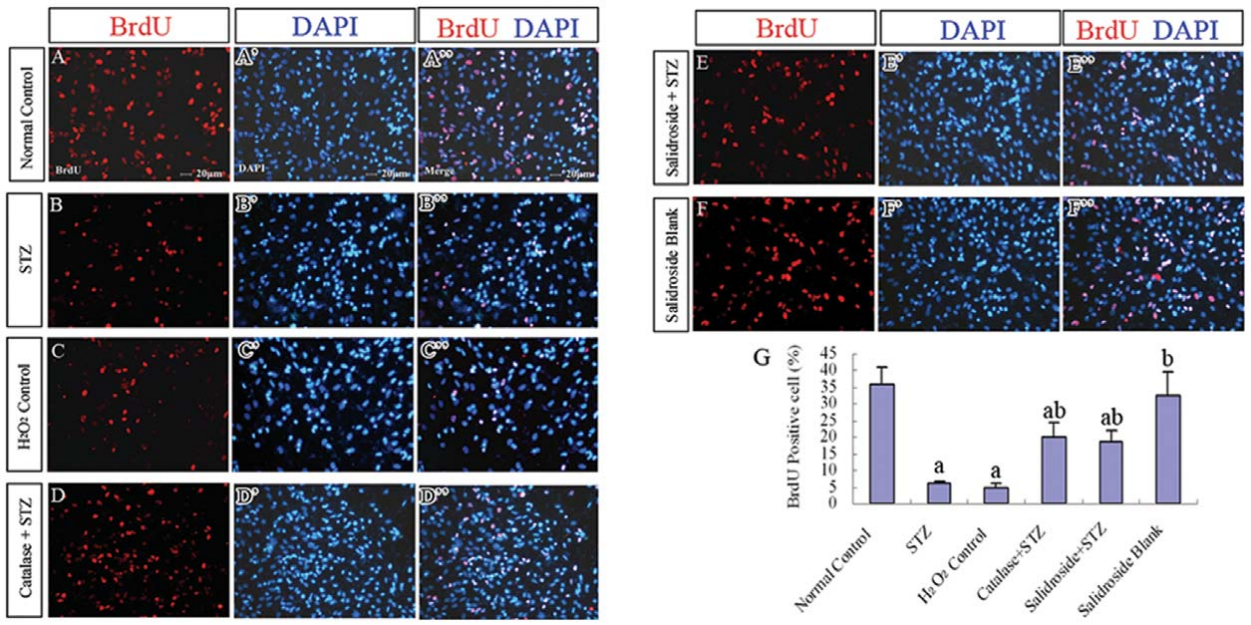
BrdU labeling in NSCs. NSCs at P2 were labeled with BrdU (10 µM) for 4 h×3 times (three times each for 4 hours), and then immunofluorescently stained for BrdU. (A–F, A′–F′, A″–F″) Representative photomicrographs of BrdU labeled cells (red, A–F) with their nuclei counterstained by the nuclear fluorescent stain DAPI (blue, A′–F′) in six experimental groups: (A-A″) Normal control group, (B-B″) STZ group, (C-C″) H_2_O_2_ control group, (D-D″) Catalase+STZ group, (E-E″) Salidroside+STZ group and (F-F″) Salidroside blank group. (G) Bar chart showing the percentages of BrdU labeled cells. Values are expressed as mean±SD. ^a^ *p*<0.05 compared with the normal control group; ^b^ *p*<0.05 compared with the STZ group.

**Figure 11:**
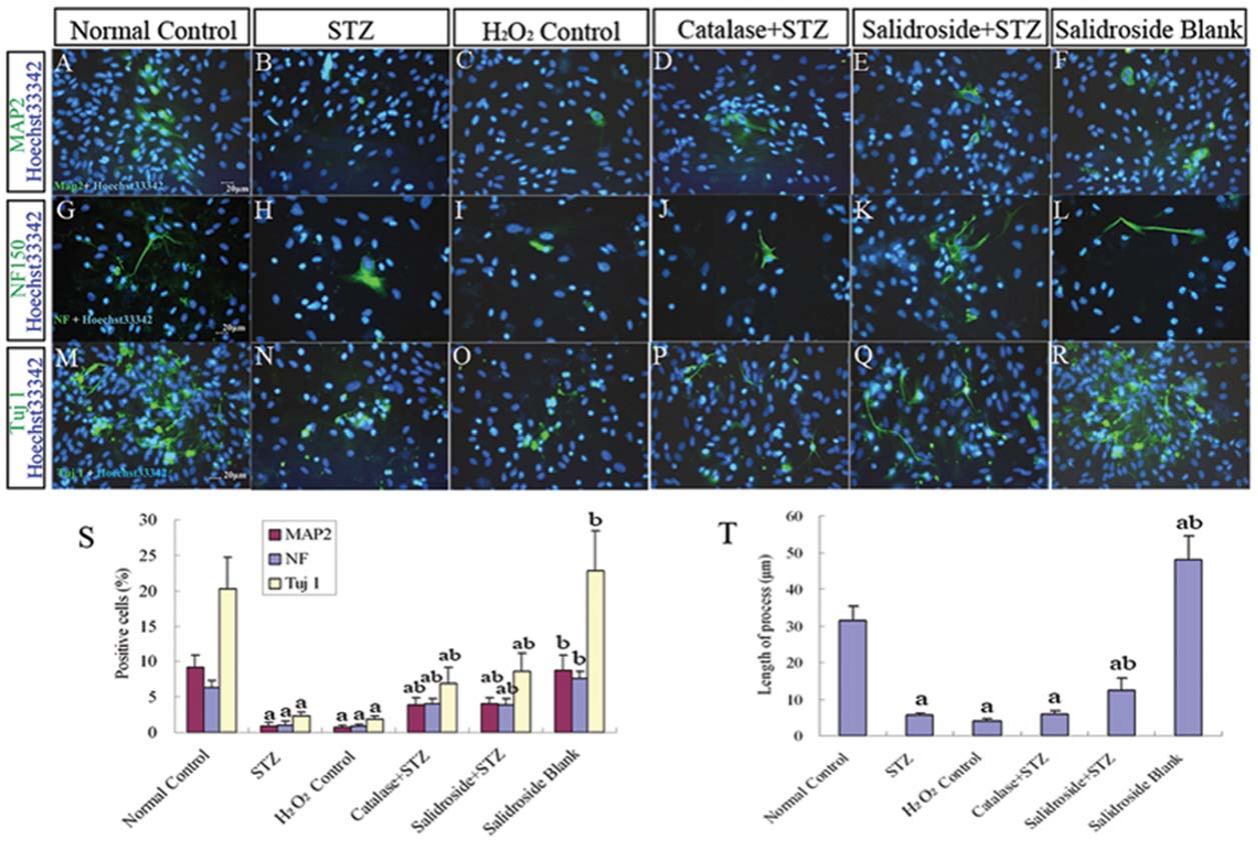
Immunofluorescence staining for MAP2, NF150 and Tuj1 in NSCs. (A–R) Representative photomicrographs of immunoreactive cells (green, nuclei counterstained with blue fluorescent stain Hoechst33342) for MAP2 (A–F), NF150 (G–L) and Tuj1 (M–R) in six experimental groups: Normal control group (A, G, M), STZ group (B, H, N), H_2_O_2_ control group (C, I, O), Catalase+STZ group (D, J, P), Salidroside+STZ group (E, K, Q) and Salidroside blank group (F, L, R). (S) Bar chart showing the percentages of immunoreactive cells for MAP2, NF150 and Tuj1. (T) Bar chart showing the average length of cellular processes from NF150 immunoreactive cells. Values are expressed as mean±SD. ^a^ *p*<0.05 compared with the normal control group; ^b^ *p*<0.05 compared with the STZ group.

The results of the MTT assay described above revealed that STZ was cytotoxic to NSCs. Next, we further examined the effects of STZ on cell survival, proliferation and neuronal differentiation of NSCs. NSCs were first incubated with 8 mM STZ for 4 h and then further incubated with the culture medium without STZ for another 8 h before the culture was examined under a light microscope. In the normal control group without incubation with STZ, NSCs attached to the culture surface, flattened and extended cellular processes (Figure 7A). When the culture was treated with STZ, many NSCs aggregated into clusters, which then detached from the culture surface (Figure 7B). Some of them managed to attach but they seldom extended elongated cellular processes. To determine the levels of necrosis and apoptosis, the cells were stained for annexin V (marker for early to intermediate stages of apoptosis) and propidium iodide (PI) (marker for late stage of apoptosis and necrosis) and also cleaved caspase-3 (marker for the advanced stage of apoptosis). The results showed significant increases of annexin V and PI positive cells in the STZ-treated group as compared to the normal control group (*p*<0.05) (Figure 8A, B, G, H). Immunostaining for caspase-3 (cleaved form) also demonstrated a significant increase in the percentage of caspase-3 positive cells in the STZ group when comparing to the normal control group (*p*<0.05) (Figure 9A-A″, B-B″, G), indicating that STZ induced an increase of apoptotic activity in the NSC culture. Then, the effects of STZ on bFGF-stimulated NSCs proliferation and FBS-induced neuronal differentiation of NSCs were studied by immunostaining for BrdU, Tuj1, MAP2 and NF150. Our results demonstrated that the percentages of cells immunoreactive to BrdU (Figure 10A-A″, B-B″, G), MAP2, NF150 or Tuj1 (Figure 11A, B, G, H, M, N, S) were all significantly lower in the STZ group than those in the normal control group (*p*<0.05) (Figures 10G and 11S). In conclusion, STZ increased cell death, decreased proliferation and reduced neuronal differentiation of NSCs, all of which might contribute to the impaired neurogenesis *in vivo* induced by the ICV injection of STZ.

**Figure 12:**
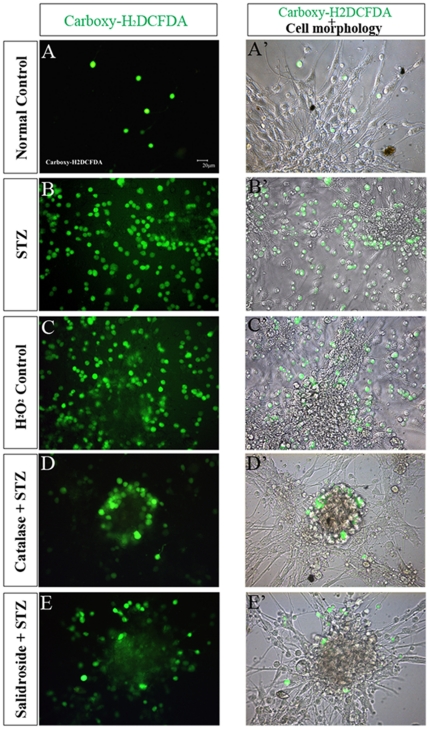
Detection of intracellular ROS with a fluorescent probe carboxy-H_2_DCFDA. Representative photomicrographs showing ROS (reactive to the green fluorescent probe carboxy-H_2_DCFDA) within the cytoplasm of NSCs (A–E) and merged images showing both ROS immunoreactivity and cell morphology (A′–E′) in five experimental groups: Normal control group (A-A′), STZ group (B-B′), H_2_O_2_ control group (C-C′), Catalase+STZ group (D-D′) and Salidroside+STZ group (E-E′).

Previous studies revealed that STZ-mediated production of hydroxyl radicals and ROS was crucial to the STZ-induced pancreatic beta cell damage *in vitro* [36]. Likewise, our study *in vivo* also found that the ICV injection of STZ resulted in the impairment of neurogenesis accompanied with an increase of oxidative stress in the hippocampus (see *In Vivo* Studies (i) and (ii)). These observations prompted us to explore whether there was an increase of cellular ROS levels in STZ-treated NSCs. Results indicated that incubation of NSCs with STZ elicited a striking increase of cellular ROS, which was immunoreactive to the green fluorescent probe carboxy-H_2_DCFDA, in the STZ group (Figure 12B-B′) as compared with the normal control group (Figure 12A-A′) (*p*<0.05, Table 1). Our findings thus implicated ROS-mediated toxicity induced by STZ in NSCs.

**Table 1:** Comparison of the intracellular ROS levels in NSCs for five groups.

Groups	RFU (Relative Fluorescent Unit) value*
(a) Normal Control	56.20±13.98
(b) STZ	1209.76±340.75
(c) H_2_O_2_ Control	1330.32±432.90
(d) Catalase+STZ	389.33±61.08
(e) Salidroside+STZ	309.32±53.09

*p<0.05, when value for (b), (c), (d) or (e) compared with value for (a); value for (d) or (e) compared with value for (b); or value for (d) or (e) compared with value for (c). One-way ANOVA. Values are expressed as means±SD.

#### (iii) H_2_O_2_ Exhibited Similar Effects on NSC Survival, Proliferation, Neuronal Differentiation and ROS Levels

To further verify that the cellular activities of STZ is mediated through ROS, we incubated NSCs with a well-known oxidant, H_2_O_2,_ in replacement of STZ. As expected, H_2_O_2_ significantly reduced cell viability (Figure 7C, G), increased necrotic and apoptotic cells (Figure 8C, G, H; Fig. 9C-C″, G) and decreased BrdU labeled cells (Figure 10C-C″, G) and MAP2, NF150 and Tuj1 immunoreactive cells (Figure 11C, I, O, S). Most importantly, H_2_O_2_ also concomitantly increased cellular ROS levels (Figure 12C-C′, Table 1), as compared to the normal control group (*p*<0.05).

#### (iv) Catalase Pre-incubation Attenuated STZ Cellular Activities

To confirm the involvement of ROS in the cellular activities of STZ on NSCs, catalase, an H_2_O_2_ scavenger, was used to rescue NSCs from STZ's effects on inducing ROS formation. Pre-incubation of NSCs with catalase markedly reduced the generation of ROS induced by STZ (Table 1, Figure 12A-A′, D-D′) and reduced necrosis and apoptosis (Figure 8D, G, H; Figure 9D-D″, G) with significant increases of viability, proliferation, and neuronal differentiation of NSCs (Figures 7D, G; 10D-D″, G and 11D, J, P, S). These observations further demonstrated that STZ exerted its cellular activities on NSCs through the generation of cellular ROS.

#### (v) Salidroside Pre-incubation Similarly Attenuated STZ Cellular Activities and Reduced ROS Levels

As for the antioxidant catalase, we also found that salidroside pre-incubation in the salidroside+STZ group, as compared with the STZ group, significantly attenuated STZ activities, which included the decrease of viability (Figure 7E, G), reduction in the percentages of annexin V and PI labeled cells (Figure 8E, G, H) and caspase-3 immunoreactive cells (Figure 9E-E″, G), increase of BrdU labeling (Figure 10E-E″, G) and increase of the percentages of MAP2, NF150 and Tuj1 positive cells (Figure 11E, K, Q, S). Our results clearly demonstrated that salidroside significantly protect NSCs from STZ-induced cellular activities.

As alluded to earlier, salidroside possesses pronounced anti-oxidative properties [33], [34], and hence it is important to know whether salidroside would reduce oxidative stress in NSCs induced by STZ. It was found that pre-incubation with salidroside resulted in a significant decrease of cellular ROS in the salidroside+STZ group as compared with the STZ group (*p*<0.05) (Table 1, Figure 12E-E′). Our results therefore strongly suggested that salidroside reduces the cellular effects of STZ on NSCs by scavenging ROS.

#### (vi) Null Cytotoxicity but Enhancing Effect of Salidroside on the Extension of Cellular Processes from NF Positive Cells Derived from NSCs

As compared to the normal control group, the group treated with salidroside only (salidroside blank group) did not exhibit significant differences in the viability assay (Figure 7F, G), annexin V and PI labeling (Figure 8F, G, H), caspase-3 immunoreactivity (Figure 9F-F″, G), BrdU labeling (Figure 10F-F″, G) and immunolocalization of MAP2, NF150 or Tuj1 (Figure 11F, L, R, S), indicating that salidroside alone did not exhibit any cellular effects on NSCs.

Although salidroside did not alter the percentages of MAP2, NF150 and Tuj1 immunoreactive cells, it was intriguing to observe that the average length of cellular processes extending from NF150 positive cells was significantly increased in the group treated with salidroside only (salidroside blank group) as compared to the normal control group (*p*<0.05) (Figure 11G, L, T). The result appeared to suggest that salidroside possesses an enhancing effect on the extension of cellular processes from differentiating neurons.

## Discussion

*R. crenulata* has been previously studied in our laboratory, and an alcohol extract from the root of this medicinal plant has been demonstrated to be effective in improving cognitive functions in the rat model of AD induced by an intracerebroventricular (ICV) injection of streptozotocin (STZ) [24]. This extract has also been shown to be able to enhance hippocampal cell proliferation in depressive rats induced by chronic mild stress [30], [37]. Other studies showed that this medicinal plant possesses potent antioxidative activities [29], [33]. With these antioxidative activities, *R. crenulata* has been regarded as being capable of protecting the nervous system from oxidative damage inflicted by free radicals, rescuing neural stem cells (NSCs) within the nervous system from apoptosis or necrosis, and hence improving neurogenesis. Our previous study has already shown that continuous consumption of the alcohol extract of *R. crenulata* (*RCE*) reduced oxidative stress in the brain and prevented decline of learning and memory abilities caused by STZ in rats [24]. However, it still remains unknown as to what ingredient of the extract induces these neuroprotective effects and through what kind of cellular mechanism this ingredient exerts these effects. In the present study, we showed that pre-treatment with the *RCE* significantly improved neurogenesis in the hippocampus of the AD rats induced by ICV injections of STZ, as evidenced by the increased numbers of BrdU and Tuj1 immunoreactive cells when compared to those in the AD rats without the *RCE* pre-treatment. Concomitant with this improved neurogenesis, a reduction of oxidative stress was also observed. We then demonstrated in our *in vitro* experiments that when NSCs isolated from the hippocampus were exposed to STZ, significant increases of intracellular reactive oxygen species (ROS) was observed. Similar increases of hydroxyl radicals and ROS in the STZ-induced damage of pancreatic beta cells *in vitro* have also been reported [36]. We further demonstrated that these increased ROS levels were closely associated with the reduction of viability, proliferation and differentiation of NSCs. These cellular changes induced by STZ could be reproduced with the potent oxidant hydrogen peroxide and suppressed with pre-incubation of the antioxidant catalase. Pre-incubation of these STZ-treated NSCs with salidroside, the main ingredient of the *RCE*, similarly suppressed these cellular changes resulting in decreases of intracellular ROS with simultaneous reduction of cell death and enhancement of cell proliferation and neural differentiation of hippocampal NSCs. Our observations therefore implicated that salidroside exerted its neuroprotective effects on STZ-induced NSC injuries via its capability of scavenging ROS. Since NSCs are thought to be responsible for the neural repair through neurogenesis and associated with learning and memory recovery following CNS injury, it is likely that *R. crenulata* improves neurogenesis *in vivo* by protecting NSCs from cytotoxic injuries, and these neuroprotective effects can be ascribed to the ROS scavenging activity of salidroside.

STZ is a glucosamine-nitrosourea compound which generates a cytotoxic metabolite capable of preferentially destroying beta cells in pancreatic islets [38]. An ICV injection of a sub-diabetogenic dose of STZ to rats has been found to cause prolonged disturbances of brain glucose and energy metabolism by desensitizing neuronal insulin receptors [39]. Subsequently, these disturbances result in decreases in choline acetyltransferase levels and increased oxidative stress in the hippocampus, leading to impairments in learning and memory [40], [41]. However, STZ has no effect on blood glucose when given by ICV injections [42], implicating that its action is independent of hyperglycemia. Recent studies found that the rat model with ICV injection of STZ is able to mimic the cellular and molecular abnormalities including hyperphosphorylation of Tau protein and senile plaque-like deposits of sporadic AD [42], [43]. In fact, the ICV injection of STZ has already been widely used as an experimental method to induce different types of neurodegenerative diseases, including AD [40], [44], in experimental animals.

Many studies indicated that salidroside, the main bioactive compound of *Rhodiola* plants, possesses anti-oxidative properties and thus contributes to the neuroprotective activities of the medicinal plant [34], [35]. Recently, the compound has been further demonstrated to be able to stimulate erythropoiesis [45], enhance hippocampal cell proliferation [30] and promote differentiation of mesenchymal stem cells into hepatocytes [46]. In our *in vitro* studies, we found that incubation with salidroside alone did not affect cell viability, proliferation and neuronal differentiation of NSCs derived from the hippocampus, but protected them against STZ-induced cell death and reduction of proliferation and differentiation by depleting the intracellular ROS. In addition to these prominent ROS scavenging activities demonstrated in this study, salidroside may also exert its neuroprotective activities through other potential pathways: (a) modulation of apoptosis-related processes by alteration of the expression of cell death related genes including down-regulation of the pro-apoptotic gene Bax and/or up-regulation of the anti-apoptotic genes Bcl-2 and Bcl-X(L) [34], [35], restoration of the mitochondrial membrane potential [47], [48] or/and suppression of cytochrome c release and caspase cascade activation [49]; (b) suppression of both the excessive entry and release of Ca^2+^ from the intracellular calcium stores, hence leading to an inhibition of the elevation of intracellular calcium levels [34], [50], [51]; and (c) inhibition of nitric oxide (NO) synthase activity and reduction of NO production by inhibition of the NF-κB-iNOS-NO signaling pathway [52]–[54]. Whether all these pathways or only some of them play roles in the neuroprotective activities of salidroside awaits further investigations.

It is worth noting that bell-shaped dose responses in neurogenesis and oxidative stress were observed in our *in vivo* experiments with the *RCE* but similar dose responses were not found *in vitro* with salidroside. Other *in vivo* observations with similar bell-shaped dose responses have also been reported [55], [56]. They found that administration with an extract of the medicinal plant of the genus *Rhodiola* at a dose of 0.1 mL improved both learning and memory in rats, while doses at 0.02 and 1.0 mL did not cause any substantial improvement. It is likely that the decline of the activity of the extract at higher doses might be attributed to the presence of some other ingredients which may interfere with neuroprotective actions of the extract. Even when the herbal extract is quantified with known active or marker compounds to achieve more consistent pharmaceutical properties, variations in the concentrations of other constituents can affect its activities and biological efficacies [57]. Conversely, we also cannot exclude the possibility that some other components of the *RCE* may also account for the neuroprotective effects of *R. crenulata*. The root of the medicinal plant *R. crenulata* contains more than 21 compounds. Besides salidroside, p-tyrosol is another important active constituent of the plant [31], [32]. Tyrosol has been relatively well studied as the main ingredient of some other dietary food such as white wine and olive oil [58]–[61], and a variety of cellular activities of tyrosol including antioxidant activity [59], [62], neural protective functions [61] and anti-inflammatory effects [60] have been reported. Hence tyrosol might also exert its neuroprotective effects through its antioxidant activity, just like salidroside. If tyrosol is also found to exhibit neuroprotective activities on NSCs, then the combined activities of salidroside and tyrosol may attribute to the protective actions of the *RCE*.

The deposits of beta amyloid protein (Aβ) and hyperphosphorylation of Tau protein in brain regions are the main pathological hallmarks of AD, and these proteins elicit the pathological cascade responsible for dementia, neuropsychiatric changes and finally death of neurons. The ICV administration of STZ also leads to an increase in the total Tau and Aβ protein [42], [43], in addition to the free radical formation [40]. It is therefore of special interest in investigating the changes of Aβ or phosphorylated Tau protein levels in the STZ-treated and *RCE*-protected rats. Studies have already indicated that Aβ might cause deterioration of the brain microenvironment which is important for neurogenesis of NSCs [63], [64]. A recent study also showed that salidroside has protective effects against Aβ-induced oxidative stress in SH-SY5Y human neuroblastoma cells [48]. To better understand the neuroprotective activities of the *RCE*, it is imperative to determine the potential protective actions of salidroside against defective neurogenesis caused by Aβ in future studies.

Intriguingly, although salidroside did not seem to be able to induce overt neuronal differentiation of NSCs, it exhibited an enhancing effect on the extension of cellular processes from some neuron-like cells derived from NSCs in this study. This suggests that salidroside might promote or expedite the maturation of newly developed neurons, although more investigations need to be carried out to confirm this observation.

Taken together, salidroside, as one of the main bioactive ingredients of *R. crenulata*, most probably acts by scavenging ROS to protect NSCs from necrosis and apoptosis, and also improve proliferation and neuronal differentiation of NSCs in the hippocampus of STZ-treated rat. All these contribute to the protective effects of the *RCE* on defective neurogenesis and cognitive impairment in AD rats. Our findings raise the possibility that the *RCE* has the therapeutic potential to improve hippocampal neurogenesis and thus treating AD.

## Materials and Methods

### Chemicals

The sources of different chemicals, reagents and assays kits are listed below: Salidroside (>98%) from Tianjin Jianfeng Natural Product R&D Co., Ltd.; Glutathione reductase (GR), reduced glutathione (GSH) and malondialdehyde (MDA) detection kits and Coomassie brilliant blue protein assay kit from Nanjing Jiancheng Bioengineering Institute (Jiangsu province, China); Sodium carboxymethycellulose (CMC-Na) from Tianjin Fuchen Chemical Reagent Factory (Jiangsu province, China); Streptozotocin, Hoechst33342 and 5-bromo-2′-deoxyuridine (BrdU) from Sigma Chemical Co., St. Louis, USA; Catalase, H_2_O_2_, 4′,6-diamidino-2-phenylindole (DAPI) and 3-(4,5-dimethylthiazol-2-yl)-2,5-diphenyl-tetrazolium bromide (MTT) from Amresco Inc.; Dulbecco's modified Eagle's medium/F12 (DMEM/F12) (1∶1), bFGF, B27 and fetal bovine serum (FBS) from GIBICO, Invitrogen Inc; Annexin V Apoptosis Detection Kit (sc-4252 AK) from Santa Cruz Biotechnology, Inc.; Image-iT™ LIVE Green Reactive Oxygen Species Detection Kit (I36007) from Molecular Probes, Invitrogen Inc. All other reagents were purchased from Sigma Chemicals and were of analytical grade unless stated otherwise.

### *R. Crenulata* Extract (RCE)

An alcohol extract of the edible *R. crenulata* root was used in this study. The *R. crenulata* used for extraction was obtained from Guangzhou Baoxing Bio-technologies Co., LTD (China), and was authenticated by a plant taxonomist. The root of the plant was first dried and ground with a grinder, and the coarse powder of the plant was then extracted with 70% alcohol twice, 2 hr each. The extract was condensed by vacuum, then collected with ethanol precipitation, and finally spray dried to yield a reddish-brown powder. The yield of the *R. crenulata* extract was about 3–5% (w/w). The concentration of the known active component salidroside in the extract was determined by HPLC and was found to be 4% w/w.

### Animals

Male Sprague Dawley rats weighing 240–260 g were used *in vivo* while *in vitro* studies were conducted on primary hippocampal neural stem cells (NSCs) prepared from newborn rats at postnatal day zero (P0). All the animals were purchased from the Experimental Animal Center of Sun Yat-sen University, Guangzhou, China. Rats were fed with standard chow diet and tap water *ad libitum*, and housed in pair in separate cages which were maintained at a temperature of 24±2°C under 50–60% relative humidity with 12 h light/dark cycles throughout the experiment. The animals were kept in the facility for at least 1 week before the experiment started. Rats were then divided into five groups as indicated in the following section. All experimental protocols and animal handling procedures adopted in the present study were approved by the Animal Care and Use Committee of Sun Yat-sen University and were consistent with the National Institutes of Health Guide for the Care and Use of Laboratory Animals.

### Grouping and Treatments In Vivo

Rats were divided randomly into five groups of 12 animals: normal control group (injected with an artificial cerebrospinal fluid which was also the solvent for STZ. For components of the fluid, see next section), STZ group (injected with STZ only), L-RCE+STZ group (pre-treatment with a low dose of 1.5 g/kg *RCE* and then STZ treatment), M-RCE+STZ group (pre-treatment with a medium dose of 3.0 g/kg *RCE* and then STZ treatment) and H-RCE+STZ group (pre-treatment with a high dose of 6.0 g/kg *RCE* and then STZ treatment). The *RCE* (*R. crenulata* extract) was diluted with 0.5% CMC-Na water solution and given orally everyday at a single dose of 2 ml by gavages for 21 days before the STZ injection (see next section). The normal control and STZ groups of rats received orally the same volume (i.e. 2 ml) of 0.5% CMC-Na solution for 21 days.

### Intracerebroventricular Injection

STZ was injected intracerebroventricularly (ICV) as described previously [40]. Briefly, rats were anesthetized with sodium pentobarbital at an intraperitoneal dose of 40 mg/kg. The head was positioned within a stereotactic frame, and the skull was exposed. Burr holes were drilled in the skull on both sides over the lateral ventricles using the following coordinates [65]: 0.8 mm posterior to the bregma; 1.8 mm lateral to the sagittal suture; and 4.0 mm beneath the surface of skull. Each rat of the STZ group and the three *RCE* pre-treatment groups was given bilateral ICV injections of 1.5 mg/kg STZ. STZ was dissolved in an artificial cerebrospinal fluid (artificial CSF: 147 mM NaCl, 2.9 mM KCl, 1.6 mM MgCl_2_, 1.7 mM CaCl_2_ and 2.2 mM dextrose), and a STZ solution of 25 mg/ml was freshly prepared on ice just before the injection. The injections of the same dose of STZ were repeated on the 3rd day after the first injection on the first day. Rats of the normal control group underwent the same surgical procedure and was injected with the same volume of the artificial CSF (the exact volume was calculated according to the body weight of individual animal) instead of STZ.

### BrdU Administration

In order to study cell proliferation and differentiation in the hippocampal dentate gyrus, rats received twelve consecutive daily intraperitoneal injections of BrdU (at 50 mg/kg body weight) during the period of the 9–20th days after ICV STZ injections. BrdU was dissolved in 0.9% NaCl and sterilized through a 0.45 µm filter. The animals were sacrificed 24 h after the last BrdU injection.

### Hippocampal Sample Preparations

#### (i) Hippocampal homogenate

Rats were sacrificed on day 21 after the first ICV injection of STZ. The entire brains were removed quickly for dissecting the hippocampus on ice. The hippocampuses were weighed and then homogenized in cold normal saline to obtain a 10% (w/v) homogenate solution. The homogenate was centrifuged at 700 g for 8 min at 4°C. The supernatant was collected, and aliquots were stored at −80°C for detection of MDA, GR and GSH and the protein assay.

#### (ii) Hippocampal tissue sections

Three weeks after the first ICV STZ injection, rats were deeply anaesthetized with sodium pentobarbital. The animals were fixed by transcardial perfusion, first with 150 ml normal saline containing heparin (5 U/mL) and then 250 ml 4% (w/v) paraformaldehyde (PFA) in 0.01 M phosphate buffered saline (PBS, pH7.4). The brains were dissected and postfixed in the same fixative overnight and transferred to 30% sucrose solution for cryoprotection. Coronal sections at 40 µm were cut in a cryostat. Every 12 coronal sections were selected, and six sections were collected from each rat for the examination of neurogenesis in the hippocampus.

### Indicators for Oxidative Stress in the Hippocampus

According to the instructions of the test kits, the amounts of malondialdehyde (MDA) and glutathione (GSH) and also the activity of glutathione reductase (GR) were measured spectrophotometrically with the thiobarbituric acid (TBA) method [66], the method of Ellman [67] and the method of Carlberg and Mannerviek [68], respectively. The concentrations of MDA and GSH were expressed as nmol/mg protein and mg/g protein, respectively. The enzyme activity of GR was quantified at 25°C by measuring the decline of NADPH at 340 nm. 1 U of GR was defined as the amount of GR in 1 g tissue protein which catalyses 1 mM NADPH oxidation per minute. Protein content was assayed with Coomassie brilliant blue by following the test kit instruction. The absorbance was measured at 595 nm using a spectrophotometer.

### Tuj1/BrdU Double Immunofluorescence Labeling

The phenotype of differentiating cells was studied with Tuj1/BrdU double immunofluorescence labeling described elsewhere [9], [69]. For BrdU detection, DNA was denatured by incubation of sections for 2 h in 50% formamide/2×SSC (0.3 M NaCl and 0.03 M sodium citrate) at 65°C. The sections were then rinsed for 5 min in 2×SSC, incubated for 30 min in 2 N HCl at 37°C and then rinsed again for 10 min in 0.1 M boric acid, pH 8.5. Double immunofluorescence labeling was performed with the mouse monoclonal anti-BrdU antibody (Sigma Chemical Co., St. Louis, USA; 1∶400) and rabbit polyclonal anti-βIII tubulin (Tuj1) antibody (Sigma Chemical Co., St. Louis, USA; 1∶60) as the primary antibodies and FITC- and Cy3-conjugated antibodies as the secondary antibodies (Jackson Immunological Research; 1∶100 for FITC, and 1∶400 for Cy3), respectively. Negative controls for BrdU labeling were performed by using sections of brain tissues from rats without receiving BrdU injection while for the negative controls for Tuj1 staining, the primary antibody treatment was replaced by incubation of PBS (solvent for the antibodies) during the staining procedure. Sections were mounted with 30% glycerin in PBS and examined under an epifluorescence microscope (Leica, Switzerland) equipped with the Leica IM50 Image Plus computer-assisted image analysis system.

### Preparation and Characterization of NSCs

NSCs were prepared from the hippocampal tissue of newborn rats at postnatal day 0 following our procedure described earlier [70]. Briefly, the entire hippocampus was dissected and dissociated in D-Hanks' balanced salt solution (HBSS). The cell suspension was centrifuged at 1000 rpm for 5 min. The pellet was then re-suspended in DMEM/F12 supplemented with B27 (2 ml/100 ml) and bFGF (20 ng/ml). The cells were plated to 75 ml culture flasks (final density: 1×10^5^ viable cells/µl). Half of the medium was refreshed every three days. Typically, the cells grew as neurospheres in suspension and were passaged approximately once per week. Throughout the study, neurospheres at the second passage (P2) were used. To confirm the neurospheres contained nestin immunoreactive cells, neurospheres were fixed with 4% PFA for 30 min at room temperature, rinsed in PBS, labeled with a monoclonal antibody to nestin (Sigma Chemical Co., St. Louis, USA; 1∶1000), a marker of neural stem cells [71], and then incubated with FITC-conjugated anti-mouse IgG (1∶100, Jackson Immunological Research, PA, USA). To examine their neuronal differentiation potential, P2 neurospheres were directly plated on poly-L-lysine-coated coverslips and grown in the differentiation culture medium (DMEM/F12+10% FBS) for seven days. After fixation with 4% PFA, cultured cells were immunostained for Tuj1 (immature neuron marker), glial fibrillary acidic protein (GFAP) (astroglia marker, [72]) and myelin oligodendrocyte specific protein (MOSP) (oligodendrocyte marker), using rabbit polyclonal anti-βIII tubulin (Sigma Chemical Co., St. Louis, USA; 1∶60), rabbit polyclonal anti-GFAP (Sigma Chemical Co., St. Louis, USA; 1∶300) and mouse monoclonal anti-MOSP (Sigma Chemical Co., St. Louis, USA; 1∶1000) antibodies as primary antibodies, respectively, followed by incubation with FITC-conjugated anti-rabbit IgG (1∶100, Jackson Immunological Research, PA, USA) and Cy3-conjugated anti-mouse IgG (1∶400, Jackson Immunological Research, PA, USA) antibodies as secondary antibodies. Negative control was performed by omitting the primary antibodies. The cell nuclei were counterstained with blue fluorescent stain Hoechst33342 at 10 mg/ml.

### In Vitro Tests

To assess the protective effect of salidroside against STZ-induced cellular activities on NSCs, three different experimental protocols were employed to determine (i) the effect of different concentrations of STZ on NSC viability, (ii) the effect of different concentrations of salidroside on NSC viability and finally (iii) the protective effect of different concentrations of salidroside against the cytotoxity induced by STZ on NSCs. In the following protocols, NSCs at the second passage (P2) were plated on poly-L-lysine-coated 96-well flat-bottomed plates at 1×10^4^ cells per well, and STZ and salidroside were dissolved in warmed DMEM/F12 at the desired concentrations just before addition to NSCs. NSCs viability was assayed by MTT.

#### (i) Effect of Different Concentrations of STZ on NSC Viability

NSCs were incubated with STZ for 4 h at concentrations of 0.5, 1.0, 2.0, 4.0, 8.0, 16.0 and 32.0 mM as previously reported [73], [74]. Control cells were untreated. Each sample test was repeated three times in five independent wells. The NSC growth inhibition ratio (P) was calculated with Formula one (see below). The half maximal inhibitory concentration (IC_50_) of STZ was calculated with Formula two (see below).
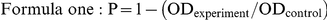


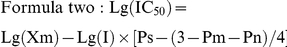



**P:** inhibition ratio; **OD_experiment_:** optical density (OD) of experiment well; **OD_control_:** OD of control well; **Xm:** maximal concentration; **I:** dilution factor; **Ps:** sum of inhibition ratio; **Pm:** maximal inhibition ratio; **Pn:** minimal inhibition ratio; **Lg:** common logarithm.

#### (ii) Effect of Different Concentrations of Salidroside on NSC Viability

NSCs were pre-incubated with salidroside for 12 h at concentrations of 0, 1, 10, 100, 250, 500, 1000 and 2000 µM, which were also previously reported [34], [35]. Each sample test was repeated three times in five independent wells.

#### (iii) Protective Effect of Different Concentrations of Salidroside against Cellular Activities Induced by STZ on NSCs

NSCs were pre-incubated with salidroside for 12 hr at concentrations of 0, 1, 10, 100, 250, 500, 1000 and 2000 µM before exposure to STZ for 4 h at the IC_50_ concentration. Control cells were untreated. Each sample test was repeated three times in five independent wells. The concentration of salidroside which exerted the maximal protective effect was used in all subsequent experiments.

### Grouping and Drug Administration in Vitro

NSCs at P2 were seeded on poly-L-lysine-coated cultured plates and cultured with the plain culture medium alone (Normal Control group), H_2_O_2_ at 1 mM [75] for four hours (H_2_O_2_ Control group), STZ for four hours (STZ group), salidroside for twelve hours (Salidroside Blank group), salidroside for twelve hours followed by a four-hour incubation with STZ (Salidroside+STZ group), or catalase at 50 µg/ml [76] for twelve hours followed by a four-hour incubation with STZ (Catalase+STZ group). The culture medium was refreshed with the plain culture medium, and NSCs were further cultured for 8 h before various assays were performed. Each experiment was repeated five times.

### MTT Viability Assay

MTT assay was used as a general indicator of cellular viability [77]. Briefly, after various treatments, 20 µl MTT (5 mg/ml) was added and the culture was incubated for additional 4 hr. The culture medium was replaced with 150 µl DMSO, and the absorbance at 490 nm was measured by a Bio-Rad reader (model 680). % Viability of NSCs was calculated with Formula three:
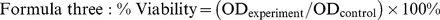



### Annexin V Labeling

An early indicator of apoptosis is the rapid translocation and accumulation of the membrane phospholipid phosphatidylserine from the cytoplasmic interface to the extracellular surface [78]. This morphological change in the plasma membrane was detected with the Annexin V Apoptosis Detection Kit according to the manufacturer's instructions (Santa Cruz Biotechnology, Inc.). In brief, NSCs were washed with the Assay Buffer and incubated with the solution of Annexin V (10 µg/ml, FITC conjugated), propidium iodide (PI, 1 µg/ml) and Hoechst33342 (1 µM) in the dark at room temperature for 15 min. Cells were mounted in the Assay Buffer and photographed with a Leica epifluorescence microscope. Normal viable cells were stained negative for both FITC conjugated Annexin V and PI while FITC-Annexin V bound to early apoptotic cells, which were not stained by PI. Cells at late stages of apoptosis and necrotic cells were stained positive for Annexin V FITC and PI. The percentages of apoptotic and necrotic cells were determined by counting the numbers of cells with Annexin V positive but PI negative (early-to-intermediate stages of apoptosis), cells with PI positive plus chromatin condensation (late apoptosis), and cells with PI positive but without chromatin condensation (necrosis). The total number of cells were counterstained with Hoechst33342.

### Caspase-3 Immunofluorescence Staining

Caspase-3, one of the key enzymes of apoptosis, was immunofluorescently detected with the rabbit anti-cleaved caspase-3 (Asp^175^) pAb (Calbiochem) as the primary antibody. After various drug treatments, NSCs were fixed with 4% PFA, rinsed in PBS, incubated in 10% (v/v) goat normal serum to block non-specific binding sites, and then incubated with the primary antibody (1∶1000) at 4°C overnight. After washes in PBS, the cells were incubated with Cy3-conjugated anti-rabbit IgG (1∶400, Jackson Immunological Research, PA, USA) at room temperature for 30 min, rinsed in PBS, then counterstained with nuclear fluorescent stain Hoechst33342 and mounted with glycerin PBS. Negative control was performed by omitting the primary antibody. Positive cells were counted under a Leica epifluorescence microscope, and their percentage was calculated.

### Detection of Intracellular ROS Using Carboxy-H_2_DCFDA

The Image-iT™ LIVE Green Reactive Oxygen Species (ROS) Detection Kit (Molecular Probes, Invitrogen Inc.) was used to assess intracellular ROS levels in NSCs. The assay is based on the reaction of ROS with the hydrolytic products of 5-(and-6)-carboxy-2′, 7′-dichlorodihydrofluorescein diacetate (carboxy-H_2_DCFDA), a reliable fluorogenic marker for ROS in live cells [79]. After diffusing into the cells, carboxy-H_2_DCFDA is hydrolyzed to the polar non-fluorescent form (carboxy-DCFH) and trapped within cells. The carboxy-DCFH is further oxidized by ROS to a fluorescent dichlorofluorescein (carboxy-DCF). After various drug treatments, ROS levels within NSCs were detected by following the manufacturer's instructions of the detection kit (Molecular Probes, Invitrogen Inc.). The cells were examined under a Leica epifluorescence microscope with the excitation source and filter for fluorescein FITC. For quantification, the cells were examined with a fluorescent plate reader (TECAN, Genios) at excitation and emission wavelengths of 485 and 535 nm, respectively. The relative fluorescent unit (RFU) of each sample was calculated (Table 1).

### BrdU Labeling

NSCs were treated with BrdU (10 µM) three times (each for 4 hr) from the time when STZ was administered. BrdU labeling was performed with BrdU/DAPI immunofluorescent double labeling method [80], [81]. After NSCs were fixed with 4% PFA, nuclei were first stained with the DNA-marker DAPI (50 µg/ml) at 37°C for 20 min, and then the DNA of cells was denatured by incubation in 2 N HCl at 37°C for 30 min. Non-specific immunoreactions were blocked with 10% normal goat serum. The mouse monoclonal anti-BrdU antibody (Sigma Chemical Co., St. Louis, USA; 1∶400) was used as the primary antibody, and Cy3-conjugated anti-mouse IgG (Jackson Immunological Research; 1∶400) as the secondary antibody. The staining procedure was the same as the caspase-3 staining described above. Negative control was performed by using NSCs without receiving any BrdU treatments. Fluorescent signals were detected under a Leica epifluorescence microscope. BrdU positive cells were counted and compared to the total number of cells as determined by DAPI nuclear staining.

### Detection for Neuronal Differentiation of NSCs

Experimental grouping was the same as above, but the treatment procedure was slightly modified for the detection of neuronal differentiation of NSCs. Neurospheres at P2 were plated on poly-L-lysine-coated coverslips and grown in the differentiation culture medium for seven days. The concentrations of various treatment agents remained unchanged. The time of exposure to STZ or H_2_O_2_ was changed to 1 h per day for 7 consecutive days. Every day, the culture medium was refreshed after STZ/H_2_O_2_ treatment. The cells were pretreated with salidroside or catalase for 4 hr before each STZ treatment. After various treatments, immunofluorescence localization of neuronal markers, namely MAP2, NF150 and Tuj1, was performed using mouse monoclonal anti-MAP2 (Sigma Chemical Co., St. Louis, USA; 1∶300), rabbit polyclonal anti-NF150 (Sigma Chemical Co., St. Louis, USA; 1∶200) and rabbit polyclonal anti-βIII tubulin (Sigma Chemical Co., St. Louis, USA; 1∶60) primary antibodies, respectively, and FITC- (1∶100) and Cy3-conjugated (1∶400) secondary antibodies (Jackson Immunological Research). Negative controls were performed by omitting the primary antibodies.

### Cell Counts and Statistical Analyses

After immunostaining, fluorescent signals were examined under a Leica epifluorescence microscope. For quantification of neurogenesis *in vivo*, six coronal sections through the dorsal hippocampus (anterio-posterior extent from 15.86 to 12.96 mm) from each animal were analyzed for BrdU-positive cells in the granular cell layer (GCL) and the subgranular zone (SGZ) of the dentate gyrus (DG). The SGZ was defined as the cellular layer of two-nuclei wide below the border between the GCL proper and the hilus [82], [83]. All BrdU positive nuclei in these selected areas were counted and the percentages of BrdU^+^Tuj1^+^ double positive cells among all BrdU positive cells were calculated. For quantification of immunoreactive cells *in vitro*, cells in each culture were counted with a 20× objective. Five visual fields were selected from the upper left, upper right, lower left and lower right corners and also the central part of the culture with a total of 25 visual fields. Data collected for each culture included the total number of cells (based on Hoechst33342 or DAPI staining) and numbers and percentages of cells immunoreactive to individual antibodies.

### Statistical Analyses

Data analyses were performed with one-way ANOVA, followed by *post hoc* comparison with the S-N-K *post hoc* test using SPSS software (Version 12.0, SPSS Inc., Chicago, IL, USA). Data were presented as mean±SD. Significance levels were set to 0.05 for all comparisons.
